# Dr Reddit? The quality and accuracy of information about IVF add-ons on Reddit

**DOI:** 10.1093/heapro/daag151

**Published:** 2026-10-14

**Authors:** Sara Attinger, Joscelyn Gan, Amandi Perera, Nikka Milani, Kim Doyle, Michelle Steeper, Jack Wilkinson, Ektoras X Georgiou, Tammy Hoffmann, Sarah Lensen

**Affiliations:** Obstetrics, Gynaecology and Newborn Health, Faculty of Medicine, Dentistry and Health Sciences, University of Melbourne, Melbourne, Level 7 Royal Women′s Hospital, Victoria 3052, Australia; Melbourne Medical School, University of Melbourne, Grattan Street, Parkville, Victoria 3010, Melbourne, Australia; Obstetrics, Gynaecology and Newborn Health, Faculty of Medicine, Dentistry and Health Sciences, University of Melbourne, Melbourne, Level 7 Royal Women′s Hospital, Victoria 3052, Australia; St Vincent's Hospital Melbourne, Melbourne, Australia; Melbourne Data Analytics Platform, School of Computing and Information Systems, Faculty of Engineering and Information Technology, University of Melbourne, Level 8, Melbourne Connect, 700 Swanston Street, Carlton, Victoria 3053, Melbourne, Australia; Obstetrics, Gynaecology and Newborn Health, Faculty of Medicine, Dentistry and Health Sciences, University of Melbourne, Melbourne, Level 7 Royal Women′s Hospital, Victoria 3052, Australia; Centre for Biostatistics, Manchester Academic Health Science Centre, Division of Population Health, Health Services Research and Primary Care, University of Manchester, Jean McFarlane Building, Oxford Road, Manchester M13 9PL, Manchester, United Kingdom; Obstetrics, Gynaecology and Newborn Health, Faculty of Medicine, Dentistry and Health Sciences, University of Melbourne, Melbourne, Level 7 Royal Women′s Hospital, Victoria 3052, Australia; Melbourne IVF, 36 Wellington Street, Collingwood, Victoria 3066, Australia; Reproductive Services, Royal Women’s Hospital, Level 7, Parkville, Victoria 3052, Australia; Institute for Evidence-Based Healthcare, Bond University, Building 5, Level 4, 14 University Drive, Robina, Queensland 4226, Gold Coast, Australia; Obstetrics, Gynaecology and Newborn Health, Faculty of Medicine, Dentistry and Health Sciences, University of Melbourne, Melbourne, Level 7 Royal Women′s Hospital, Victoria 3052, Australia

**Keywords:** Reddit, IVF add-ons, social media, online, peers

## Abstract

IVF add-ons are optional tests, treatments or procedures offered in addition to standard IVF, often at additional cost and with uncertain evidence of benefit. In a commercialized fertility sector, patients must make complex reproductive decisions while navigating variable clinical information, online advice and peer-to-peer narratives. This study examined Reddit as a digital health environment shaping access to information and decision-making about IVF add-ons. Posts and comments from the r/IVF subreddit were extracted from 14 October to 13 November 2024, filtered using IVF add-on keywords, and manually reviewed for eligibility. Eligible content included anecdotes, claims about benefit or lack of benefit, and/or recommendations to use or avoid an add-on. Accuracy of claims was independently assessed by two researchers against evidence-based sources, including Cochrane reviews. Of 32 420 posts and comments screened, 1498 were eligible, comprising 2011 relevant statements: anecdotes (*n* = 1414), claims (*n* = 250), and recommendations (*n* = 347). The most frequently mentioned add-ons were preimplantation genetic testing for aneuploidy, aspirin, antioxidants, acupuncture, and growth hormone. Anecdotes more commonly described success than failure (64% vs 36%). Most claims asserted benefit (161/250, 64%), and most benefit claims were unsupported or contradicted by robust evidence (111/161, 69%). Recommendations overwhelmingly encouraged add-on use (280/347, 81%), despite limited supporting evidence. Online forums can provide valuable peer support, but may also amplify unsubstantiated benefit claims and commercially aligned treatment norms. Accessible, independent, evidence-based digital resources are needed to strengthen reproductive autonomy and informed decision-making in women’s reproductive health.

Contributions to Health Promotion

- Reddit content about IVF add-ons frequently promotes interventions with limited evidence of benefit.
- Anecdotes and recommendations strongly favoured add-on use
- Most claims about benefit of add-ons were unsupported or contradicted by robust evidence.
- Accessible, independent, evidence-based digital resources are needed to support informed decision-making in commercialized IVF care.

## Background

Many IVF optional treatments or ‘add-ons’ are available and used with the aim of increasing the chance of a successful outcome. These treatments are non-essential procedures, techniques, or medicines which can be added to standard IVF protocols. Examples include endometrial scratching, acupuncture and preimplantation genetic testing for aneuploidy (PGT-A). Most IVF add-ons are not supported by good-quality evidence of their safety or that they increase the chance of live birth (Armstrong *et al*. 2019, Lensen *et al*. 2019, Lensen *et al*. 2026) Despite this, IVF add-on use is widespread in many settings with >70% of patients reporting use of at least one (Lensen *et al*. 2021, HFEA 2025).

In a highly commercialized fertility sector, patients are required to make complex, high-cost reproductive decisions while navigating variable clinical information, online advice and peer-to-peer narratives. The internet and social media have become a key source of health information for many consumers, allowing people to readily access and create health-related content (Kickbusch and Holly 2023). Most IVF patients seek information online to inform treatment decisions, including via social media and online forums such as Reddit (Afroz *et al*. 2026) Reddit is a large, publicly accessible, open-source online discussion platform organized into topic-specific communities known as ‘subreddits’. Users post questions, share experiences, and comment on others posts, typically anonymously, with content ranked by community voting in the form of up-votes and down-votes, posts with more up-votes are more visible to other users. The r/IVF subreddit is highly active with over 76 000 members, and 209 000 weekly visitors. Digital forums may provide valuable peer support, but they may also shape perceptions of benefit, risk and normal practice in ways that influence patient expectations and treatment decisions. As is the nature of these online forums, the potential for misinformation—false, inaccurate, or misleading information that is presented and shared as though it were true, intentionally or unintentionally—is substantial (eSafety Commissioner 2026). For example, misinformation about IVF add-ons can include inaccurate claims about efficacy, safety, or the certainty of the evidence for an optional extra treatment.

Reddit differs from other social media platforms in multiple ways. It is an open-access platform; registered users can post and comment without verification of their identity or expertise, while subreddit content can generally be viewed without creating an account or logging in (Reddit 2024a). Users post under pseudonyms, which may encourage candid disclosure of experiences but also makes it difficult to verify who is providing information—whether current or former patients, clinicians, clinic representatives, or product marketers. Content is subject to moderation by volunteer, subreddit-specific moderators, whose role is generally to enforce community guidelines (e.g. removing spam or abusive content) rather than to verify the clinical accuracy of claims made in posts or comments (Reddit 2024b). As a result, Reddit can host a wide range of both accurate, evidence-based information and unverified or misleading claims side by side, with little to distinguish one from the other. Reddit's culture of anonymity also distinguishes it from identity-driven platforms such as Instagram and Facebook, likely reducing influencer-style promotion. At the time of our study, r/IVF's moderation rules prohibited selling, reinforcing its character as a peer-to-peer rather than commercial space.

Despite substantial use of Reddit by people undergoing IVF, the quality and accuracy of information shared about IVF add-ons on the platform remain unclear. This study examined Reddit as a digital health environment shaping access to information and decision-making about IVF add-ons, and evaluated the quality and accuracy of add-on-related content on r/IVF.

## Materials and methods

As the study aimed to describe the quality of content on Reddit about IVF add-ons and evaluate claim accuracy, we performed a hybrid conventional content analysis with a directed approach. Posts and associated comments (hereafter referred to as ‘posts’) from the ‘r/IVF’ forum between 14 October and 13 November 2024 were extracted using the Reddit Application Programming Interface using Python’s Praw package and filtered for keywords relating to IVF add-ons (see Supplementary file).

Extracted posts were manually reviewed for eligibility and included if they contained at least one of the following content categories in relation to one or more specific add-ons: personal anecdotes about use/explicit non-use (e.g. ‘I had acupuncture around my transfer and am now 14 weeks’), claims about benefits (or lack of) (e.g. ‘PGT-A is proven to increases your chances’), recommendations to use/not use (e.g. ‘If I were you, I would add omnitrope and coQ10’). We used a predefined coding framework including definitions and examples to ensure consistency, and categories were further refined inductively into subcategories to capture nuances in user perspectives (see Supplementary file). Mentions of multiple add-ons were counted separately.

All posts were assessed for eligibility and coded by at least one team member (S.A., J.G., A.P., and N.M.), with a random subset of 134 posts (2%) double-coded by a second reviewer to ensure consistency and agreement. Claims were categorized as claiming benefit or no benefit (the latter including no evidence of benefit, limited benefit, and claims that the add-on may cause harm). Accuracy was assessed by two researchers (S.A. and M.S.) independently against trusted sources, and any conflicts were resolved by a third coder (S.L.) (ESHRE Guidelines, Cochrane, Evidence-based IVF) (Lundin *et al*. 2022, Lensen *et al*. 2026) Reddit posts can be up-voted or down-voted by users; these engagement metrics were also extracted and analysed descriptively.

Ethics approval was obtained from the University of Melbourne (2025-29576-71533-5). In accordance with this ethics approval, selected direct quotes have been included to illustrate differences in the accuracy and quality of information identified. However, potentially identifying details, including names of individuals or places (other than references to external publications such as scientific studies), as well as usernames, have been removed.

## Results

Of 32 420 extracted posts, 6723 contained at least one relevant keyword and were screened for eligibility (Fig. 1). Overall, 1498 posts were included after excluding 5225 without an anecdote, claim or recommendation. As posts could contain multiple relevant items, 2011 statements were analysed.

**Figure 1:**
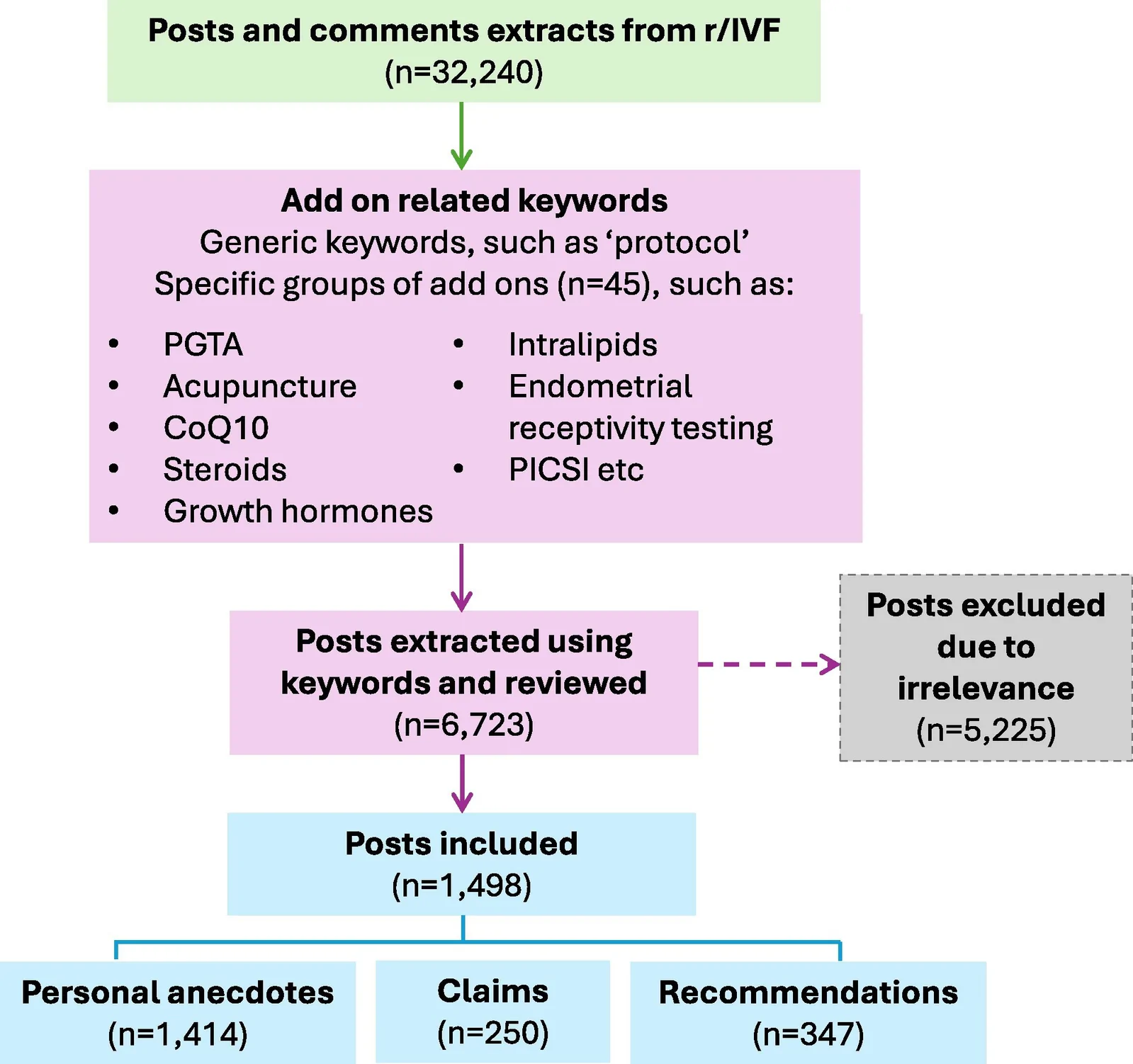
Flow diagram of post-screening and inclusion.

Thirty-one different IVF add-ons were mentioned. The most frequent were PGT-A (964, 48%), antioxidants (216, 11%), aspirin (195, 10%), acupuncture (162, 8%), and growth hormone (111, 6%). Included posts contained personal anecdotes about use or non-use (*n* = 1414), recommendations to use/not use add-ons (*n* = 347), and claims about benefit or lack of benefit (*n* = 250).

### Anecdotes

Of 1414 anecdotes, half (697, 49%) reported their IVF outcomes in relation to add-on use or non-use. There were more anecdotes reporting treatment success than failure (64% vs 36%). This was regardless of whether the anecdote described use or non-use of an add-on (Table 1). Among the remaining anecdotes that mentioned add-ons without reporting outcomes, over half (53%, 381) referred to PGT-A (e.g. ‘I got 2 euploids’).

**Table 1:** Summary of results.

Included posts	Count (% of category)	Illustrative quotes
Anecdotes	1414 (100%)	
Passing anecdote	717 (51%)	‘I got 6 euploid embryos from that batch at age 34’ (PGT-A)
Outcome based anecdotes	697 (49%)	–
Reporting add-on use	605 (87%)	–
Success	386 (64%)	‘I used it during stims—it was like a miracle for me. 5 retrievals at age 39 = 3 euploid, 1 retrieval at age 40 with Omni during stims = 3 euploid’ (growth hormone)
Failure	219 (36%)	‘just added tru Niagen for my last two cycles, both failed’
Reporting add-on non-use	41 (6%)	–
Success	27 (66%)	‘We didn’t do the PGT-A testing… I’m currently 29 weeks with one of those untested embryos and all is going well’
Failure	14 (34%)	‘1st transfer was successful without aspirin but ended in missed miscarriage’
Neutral anecdote	51 (7%)	‘I had a successful FET without aspirin. After a mmc with a subsequent transfer, I took aspirin…That one was also successful’
Claims	250 (100%)	
Benefit	161 (64%)	
Supported	13 (8%)	‘A euploid embryo has a significantly higher chance of a viable pregnancy because its chromosomal normal’
Partially supported	37 (23%)	‘There are studies on PGT[A] outcomes being better’
Unsubstantiated	79 (49%)	‘There are reasons to think Niagen might help’
Contradicted	32 (20%)	‘I’ve been advised by my clinic to take it every night continuously as it stops clotting when the embryo tries to implant’ (aspirin)
No benefit, limited benefit, disbenefit or harm	89 (36%)	
Supported	57 (64%)	‘I’d be so worried that they could discard an embryo that was viable. This can happen’
Partially supported	20 (22%)	‘there are many other things used in IVF that are not rigorously validated, like embryo glue’
Unsubstantiated	10 (11%)	‘the margin of error is just too high. For something so ‘established’, it's insane to me that they are one out of every four of their findings is incorrect, not to mention the high rate of false negatives’ (PGT-A)
Contradicted	2 (2%)	‘I asked my RE about embryo glue and he said it doesn't make a difference’
No source provided	237 (95%)	–
Recommendations	347 (100%)	
Invited	267 (77%)	–
Recommend to use	204 (76%)	‘Please consider taking Tru Niagen and NAC with a high dose prenatal and extra vitamin D it really helped my egg quality and you can take it all throughout pregnancy to help prevent miscarriage’
Neutral^a^	51 (19%)	‘Have you tried changing anything up between rounds? Like your clinic, ICSI, zymot, omnitrope, a different protocol, etc.?’
Recommend against	12 (5%)	‘I wouldn’t waste your time and money on a vaginal biome test’
Unsolicited	80 (23%)	
Recommend to use	76 (95%)	‘Wanted to see if you are considering adding Omni to your protocol next cycle to increase euploid rates?’ (growth hormone)
Recommend against	4 (5%)	‘If I was to go through another ER now even though I'm older (40 years old) I would not PGT-A-test any. True, you want to avoid miscarrying but miscarriages seem to happen regardless and meanwhile you're never giving a chance to some blastocysts that might self-correct themselves or simply having been mislabeled’
Recommend to use total	280 (81%)	–
Recommend against total	16 (5%)	–
Neutral total	51 (15%)	‘I can’t say whether or not the acupuncture made a difference, but I don’t think it hurts to try’

^a^Invited recommendations that expressed equivocation or ambivalence were classified as ‘neutral’. The same recommendations, where unsolicited, were deemed to be ‘positive’ due to their potential suggestiveness. Hence, no unsolicited recommendations were coded as ‘neutral’.

Most (68%) anecdotes were in response to posts from other users specifically soliciting stories of add-on use (e.g. ‘Has anyone tried growth hormone?’). Invited anecdotes were more positive than negative (320, 69% vs 105, 23%); whereas, unprompted anecdotes were more often negative (141, 60% vs 80, 34%).

### Claims

Of 250 included claims, 64% claimed benefit, and 36% claimed no benefit (Table 1).

Over half of the claims related to the effect on specific outcomes (141, 56%), such as egg/sperm quality (41, 16%) and miscarriage risk (32, 13%), while others were general claims of benefit or harm (109, 44%) (e.g. ‘PGTA is great and wouldn’t be offered unless it was proven’).

When comparing claims against evidence-based sources, most claims of benefit were unsubstantiated (49%) or contradicted by evidence (20%) (Table 1). Claims about limited evidence or harm were primarily supported (64%) or partially supported by evidence (22%). Some users expressed frustration at the lack of robust evidence for add-ons, with one user stating ‘It’s one of the worse parts of this IVF process: you’re never sure if ANYTHING helps or not. It’s such a mystery. Does genetic testing help my overall chances? It depends, who knows, it may. Endometrial biopsy? It’s not clear. Supplements? There’s not real evidence. Growth hormone? Inconclusive. Embryo glue? Debatable. AAAAGHHH! I could go crazy’.

Claims that were supported or partially supported by evidence had more up-votes on average than posts which were unsubstantiated or contradicted (mean (SD) votes 5.6 (8.9) vs 2.9 (4.4); median (IQR) votes 3 (5.0) vs 2 (2.0)) (Table 2).

**Table 2:** Summary of Reddit metrics and claim accuracy.

Claim accuracy rating	Counts	Average net up-vote/down-vote
Contradicted claims	34	3.2
Unsubstantiated claims	89	2.8
Partially supported claims	57	6.4
Supported claims	70	5.0
All claims	250	4.26

### Recommendations

Of 347 recommendations, most were responding to requests for advice (e.g. ‘I'm 35, should I unfreeze my remaining two embryos and do PGTA?’ (77%)) and most encouraged use of add-ons (81%). Unsolicited recommendations were fewer (23%) and mostly (95%) positive and encouraging of use.

## Discussion

Social media platforms such as Reddit are widely used by patients to inform treatment decision-making; more than 60% of IVF patients report using social media for IVF-related information and more than 40% specifically use Reddit (Afroz *et al*. 2026). In our sample alone, 6723 of 32 420 posts (21%) related to IVF add-ons, illustrating the volume and prominence of IVF add-on information sharing among patients online. This study—the first to systematically assess IVF add-on information accuracy on Reddit—found most posts expressed positive views towards add-ons.

Anecdotal content predominated, with frequent incidental references to PGT-A embedded in routine descriptions of cycle progress (e.g. ‘euploids in the freezer’). This pervasive normalization may reinforce perceptions of PGT-A as standard practice within the IVF community, despite global uncertainty and controversy about its true utility in IVF (Lensen *et al*. 2025).

We also observed a disconnect between experiences and recommendations: although claims and anecdotes were mixed, recommendations were disproportionately positive. This may reflect a ‘try everything’ mentality shaped by the high emotional stakes of infertility, perceptions that add-ons are low risk, and a desire to support peers by encouraging all possible avenues of hope (Perrotta and Hamper 2021, Armstrong *et al*. 2023, Lensen *et al*. 2023).

Consistent with previous reports of variable accuracy in online health information (Lensen *et al*. 2021, Suarez-Lledo and Alvarez-Galvez 2021, Daram *et al*. 2025) misinformation was common, particularly among claims of benefit, which were often unsupported or contradicted by evidence. Although misinformation about add-ons was prevalent, there was substantial variation in the quality of information. Some users explicitly highlighted the lack of proven benefits for specific add-ons, and accurate claims generated greater positive engagement (up-votes) than inaccurate ones (Table 2). A small subset of users demonstrated advanced, expert-level understanding of IVF add-ons and their evidence base, engaging in detailed, nuanced, and technically sophisticated discussions about them.

Importantly, the predominantly positive views identified in this study are unlikely to be explained by commercial promotion. The r/IVF community restricts commercial and self-promotional content, and paid advertisements displayed separately by the Reddit platform were not included in our dataset. The positive discourse observed therefore appears to arise predominantly from peer-to-peer discussion and patients’ own experiences and recommendations.

The variability in the accuracy of information online (Lensen *et al*. 2021, Daram *et al*. 2025) and the evolving evidence for many IVF add-ons (Lensen *et al*. 2026) highlights the need for accessible, impartial, evidence-based resources to support patient decision-making. Two prominent examples are the HFEA traffic-light system (Human Fertilisation and Embryology Authority 2022) and the University of Melbourne’s Evidence-Based IVF platform, the latter of which has been shown to improve patient comprehension and information satisfaction and reduce willingness to use unproven IVF add-ons (Afroz *et al*. 2026). However, the availability of high-quality evidence-based resources may not remove patients’ desire to seek information through social media. Peer-to-peer spaces such as Reddit may fulfil functions that formal resources are not designed to provide, particularly connection, relatability and social support. Much of the discussion identified in our study involved patients sharing experiences of add-on use and availability, and seeking support from others during what can be an isolating treatment journey. However, reliance on anecdotal experiences presents an important challenge. Our previous research indicates that patients can place substantial weight on individual stories of treatment success and may interpret temporal associations between an add-on and a successful outcome as evidence of causation (Lensen *et al*. 2023). Patients may actively seek such stories because they provide hope in the context of substantial uncertainty, making anecdotal information particularly compelling (Wrigley *et al*. 2023). Efforts to improve health literacy should therefore extend beyond simply providing accurate information, to helping patients understand, in accessible and relatable ways, why individual experiences cannot establish treatment effectiveness. The objective need not be to discourage engagement with peer communities, but to support patients to use these spaces alongside high-quality evidence-based resources, enabling experiential information and social support to complement, rather than substitute for, reliable evidence. Additionally, clinicians, scientists and health organizations have an important role in making high-quality evidence more visible and engaging within the online spaces where patients are already seeking information. This might include participating in forum discussions, hosting ‘Ask Me Anything’ sessions on Reddit, or creating accessible evidence-based content for other social media platforms. Reddit is only one part of a much broader digital information environment, and health organizations may need to engage more proactively across multiple platforms to ensure that trustworthy information is available in formats that are timely, relatable and easy to find. Rather than expecting patients to seek out conventional evidence sources, a more effective approach may be to bring reliable evidence into the online communities and social media spaces where health decisions are increasingly being shaped.

Engagement with Reddit is therefore not inherently problematic if patients are also consulting reliable evidence-based sources when making treatment decisions. However, the extent to which these information sources overlap is currently unclear. Patients who engage with Reddit may also consult resources such as the HFEA or Evidence-Based IVF, or they may rely predominantly on peer-generated content. Understanding how patients move between these different information environments, the relative weight they give to formal evidence and lived experience, and how these sources are integrated into treatment decision-making represents an important area for future research (Perrotta and Hamper 2021, Armstrong *et al*. 2023, Lensen *et al*. 2023). Although this study focused on Reddit, we recognize that the online information ecosystem is rapidly evolving, with patients increasingly using search engines and generative AI tools to seek health information. Nevertheless, Reddit remains highly active; we identified substantial ongoing discussion, and r/IVF currently reports approximately 10 000 weekly posts. Importantly, generative AI and search tools increasingly surface and cite Reddit content directly, potentially extending the reach of both accurate and inaccurate peer-generated information well beyond the original subreddit.

### Limitations

We analysed a snapshot of Reddit posts, which may not fully represent longer-term trends in IVF add-on discourse. Interpretation of post meaning may not always fully reflect author intent. While the list of key search terms was reviewed by a team of researchers and clinicians, it is possible that some lay-terms for add-ons may not have been captured. We did not measure the influence of Reddit use on individual treatment decisions. Reddit is international but predominantly anglophone, and user anonymity precluded assessment of demographics or representativeness. While we could not verify the authenticity of posts as some may have been generated by bots or non-patients, such content forms part of the information environment encountered by real users.

## Conclusion

Reddit is a prominent source of information and peer support for people undergoing IVF, but the quality and accuracy of information about IVF add-ons is overly positive. Given the persuasive power of patient anecdotes and the scale of engagement on such forums, there is a clear need for evidence-based resources to better support informed decision-making in fertility care.

## Supplementary Material

daag151_Supplementary_Data

## Data Availability

The data were derived from publicly available information on the ‘r/IVF’ Reddit page (https://www.reddit.com/r/IVF/). The content analysis coding framework is provided in the supplementary files, the extracted data and coding is available upon request.
