# Supplementary material for: Dr Reddit? The quality and accuracy of information about IVF add-ons on Reddit
Source: Health Promot Int. 2026 Oct 14;41(5):daag151. doi: 10.1093/heapro/daag151 (PMC13665465; doi:10.1093/heapro/daag151)
Supplement: Supplementary file 1 [file daag151_supplementary_data.zip › Reddit-SuppFile2-CodingFramework-Main.pdf]

0.Inclusion	1.Effectiveness					2.Anecdotes		
0.Include?	1.1.Add on	1.2.Strength of claim	1.3.Claimed effect on	1.4.Incl evidence?	1.5.Accuracy (ESHRE)	2.1.Add on	2.2.Invited?	2.3.Anecdote type
Exclude	[free text]	Claim benefit - strong	Live birth (LBR)	Mentions study	Supported	[free text]	Invited	Passing anecdote
Include		Claim benefit - moderate	Egg/sperm quality	Links to external source	Partially Supported		Unsolicited	Passing anecdote success
		Claim benefit - weak	Reducing miscarri	None	Unsubstantiated			Passing anecdote failure
		Claim no evidence of benefi	Implantation	Clinical advice	Contradicted			Implying success
		Claim no benefit	*Not specified*	*Mentions research generally*				*Implying success with caveat*
		Claim limited benefit						*Attributing success*
		Claim of harm or disbenefit						*Neutral anecdote*
								Implying failure
								Implying failure with caveat
								Attributing failure
								Implying success without addon
								Implying failure without addon
								Attributing success without addon
								Attributing failure without addon

	3.Recommendations					***4.Sharing links***		
2.4.Highest point of success	3.1.Add on	3.2.Invited?	3.3.Invited-open or specific?	3.4.Invited-for against or netural?	3.5.Unsolicited-for or against	4.1.Add on	4.2.Source type	4.3.Article (Author Year)
Not specified	[free text]	Invited	Open	For/positive	For/positive	[free text]	Review-Cochrane	[free text]
No implantation/Failed pregnancy test		Unsolicited	Specific	Against/negative	Against/negative		Review-Other	
Implantation/Chemical/Biochemical				Neutral			Guideline	
Clinical pregnancy/Pregnancy							HFEA website	
Birth							*Trial*	
Ongoing (pregnancy)							*Observational study*	
Ongoing (TWW)							*Broken link*	
Blastocyst							*Non-study link*	
Ongoing (pre-transfer)								

5.Safety	
5.1.Add on	5.2.Negative Side effect
[free text]	[free text]
