# Supplementary material for: Dr Reddit? The quality and accuracy of information about IVF add-ons on Reddit
Source: Health Promot Int. 2026 Oct 14;41(5):daag151. doi: 10.1093/heapro/daag151 (PMC13665465; doi:10.1093/heapro/daag151)
Supplement: Supplementary file 1 [file daag151_supplementary_data.zip › SuppFile1_Reddit_searchterms_260922.docx]

**Dr Reddit? The quality and accuracy of information about IVF add-ons on Reddit Supplementary File 1: List of keywords**

Acupuncture; Meridian; Trigger point; Moxibustion; Moxibustion;

Add on*; Add-on*; Addon*; Adjuvant*;

Antioxidant; Anti-oxidant; Vitamin; Selenium; Folic acid; Folate; Menevit; Glutathione; Carnitine; Carnitine; Ascorbic acid; Zinc; Fatty acid; Oil; Plant extract; tocopherol; Ubiquino*; coenzyme; Q10; multivitamins; cysteine; pycnogenol; Melatonin; radical scavengers; carotene; omega; arginine; Quercetin; Flavonoid; Riboflavin; Lutein; Lipoic acid; Diet* supplement; nutritional supplement; micronutrient; multivitamin; Nutraceuticals; Chromium; Inositol; Pentoxifylline; Astaxanthin; Lycopene; docosahexaenoic acid; magnesium;

Anti-tumour necrosis factor; Humira; TNF;

Artificial oocyte activation; AOA; Egg activat; Chemical* activat*; activation rate; ionomycin; piezoelectric stimulation; calcium ionophore;

Artificial sperm activation; Sperm activat*; ASA; SpermMobil; PTX; pentoxifylline; theophylline;

Assisted Hatching; AH; Hatching; drilling; laser; Tyrodes; thinning;

Aspirin;

Chinese medic*; TCM; Herb;

Duostim; double stim; Shanghai protocol;

Elective freeze all; Freeze-all; Freeze all;

EmbryoGlue; Glue; Embryo Glue; Hyaluron;

Endometrial receptivity testing; Endometrial biopsy; ERA test; Personalised transfer; Personalized transfer;

Endometrial scratch*;

Growth factor;

Hysteroscop*; uterine cavity assessment; endometrial polypectomy; endometrial polyps; endoscop*;

Indomethacin;

Immune protocol; Immune; Bondi protocol; Colorado protocol;

Intralipid; *lipid; Immunoglobulin; IVIG;

Steroid*; Glucocorticoid; Prednisolone; Prednisone; Pred; Dexamethasone; ACTH;

Natural killer cells; NK cells; killer-cell;

LIT; leucocyte immunization; leucocyte immunisation; LIT; rh-LIF; Tacrolimus;

Immune test*; Regulatory T cells; Regulatory T-cells; Treg; Th1; Th2; Cytokine; HLA; KIR;

GCSF; Granulocyte; G-CSF;

Hydroxychloroquine;

Intracytoplasmic morphologic sperm injection; IMSI;

Intrauterine culture; Culture device;

Intrauterine peripheral blood mononuclear cell; PBMC;

In vitro activation; IVA;

In vitro maturation; IVM;

Magnetic-activated cell sorting; Magnetic activated cell sorting; MACS;

Metformin;

Microfluidic*;

Mitochondrial DNA; mtDNA; Mitochondrial replacement;

Ovarian stimulat*;

Physiological intracytoplasmic sperm injection; PICSI; piezo ivf; sperm slow; ha-binding; ha binding;

Platelet rich plasma; PRP;

Pre-implantation genetic testing; Pre implantation genetic testing; PGT-A; PGTA; PGT; niPGT; PGS; Aneuploid*; Euploid*; Chromosome*; Mosaic; “embryo test*”; Genetic test*; Preimplantation genetic screening; PGD;

Time-lapse; Time lapse; Timelapse; TLI; incubation; incubator; Eeva; Primo Vision; Embryoviewer; Embryoscope; sequential embryo;

Seminal plasma; Seminal fluid; intracervical insemination;

Sildenafil;

Sperm DNA; Sperm oxidative stress measurement; SDF; terminal deoxynucleotidyl transferase-mediated dUTP-biotin; nick end labelling; TUNEL; in situ nick translation assay; ISNT; sperm chromatin dispersion test; SCD; Comet assay; SCSA; sperm chromatin structure assay;

Stem cell*;

Testosterone; testosterone; DHEA; DHEA; Dehydroepiandrosterone; Dehydrogesterone; testosterone cream; testosterone gel; testosterone patch;

Transfer media; hyaluronic;

Uterus flush; hCG; uterine flush; infusion*
